# When Subterranean Termites Challenge the Rules of Fungal Epizootics

**DOI:** 10.1371/journal.pone.0034484

**Published:** 2012-03-28

**Authors:** Thomas Chouvenc, Nan-Yao Su

**Affiliations:** Department of Entomology and Nematology, University of Florida, Fort Lauderdale, Florida, United States of America; University of Leeds, United Kingdom

## Abstract

Over the past 50 years, repeated attempts have been made to develop biological control technologies for use against economically important species of subterranean termites, focusing primarily on the use of the entomopathogenic fungus *Metarhizium anisopliae*. However, no successful field implementation of biological control has been reported. Most previous work has been conducted under the assumption that environmental conditions within termite nests would favor the growth and dispersion of entomopathogenic agents, resulting in an epizootic. Epizootics rely on the ability of the pathogenic microorganism to self-replicate and disperse among the host population. However, our study shows that due to multilevel disease resistance mechanisms, the incidence of an epizootic within a group of termites is unlikely. By exposing groups of 50 termites in planar arenas containing sand particles treated with a range of densities of an entomopathogenic fungus, we were able to quantify behavioral patterns as a function of the death ratios resulting from the fungal exposure. The inability of the fungal pathogen *M. anisopliae* to complete its life cycle within a *Coptotermes formosanus* (Isoptera: Rhinotermitidae) group was mainly the result of cannibalism and the burial behavior of the nest mates, even when termite mortality reached up to 75%. Because a subterranean termite colony, as a superorganism, can prevent epizootics of *M. anisopliae*, the traditional concepts of epizootiology may not apply to this social insect when exposed to fungal pathogens, or other pathogen for which termites have evolved behavioral and physiological means of disrupting their life cycle.

## Introduction

An epizootic within an insect population is defined by a sudden increase of diseased individuals [1], usually resulting in massive die off of insects due to a virulent infectious pathogen [2]. The concept of the epizootic in insects was first reviewed by Steinhaus [3] in order to explain the patterns of occurrence of infectious diseases in insect populations, which helped to establish a theoretical framework for implementation of biological control agents against insect pests [4]. One of the fundamental concepts of epizootiology is the transmission pathways adapted by a pathogen to ensure its own survival [1], because the mechanisms of pathogen transmission determine its impact on the host population and the spread of the disease [5]. Thus, in a susceptible insect host population, a pathogen relies on its ability to self-replicate and disperse within the population to trigger an epizootic wave, which has been considered an important characteristic for implementation of biological control [1]. However, practical implementation of entomopathogens as biological control agents remains challenging [6], [7] especially against soil insects due to their cryptic habitat [8]. This is particularly true when dealing with social insects [9]. For instance, the use of entomopathogens as potential biological control agents against termite pest species has been heavily investigated (more than 200 studies) within the past 50 years, but there remains no successful field implementation [10].

Social insects are known to have evolved disease resistance mechanisms within a colony to prevent the occurrence and spread of infectious diseases [11], [12]. Ants have received particular attention as a model for characterizing various aspects of their disease resistance mechanisms against the fungal entomopathogen *Metarhizium anisopliae* [13]–[17]. Termites have also evolved a range of behavioral, physiological, and immunological mechanisms (for review see [9], [18]) that result in an efficient individual and social immunity [19] against the transmission of pathogens within the termite nest.

The high densities of termites within nests favor contact among all individuals of the colony which led to the assumption that it would be easy to introduce an infectious fungal agent to trigger an epizootic as a control method for termite pest species [20]. However, it has recently been shown that this assumption was incorrect [9] because the conditions inside the termite nest did not necessarily favor the survival and replication of fungal entomopathogens [21]–[23]. Subterranean termites are capable of reducing fungi density in their nest through a complex interaction of defense mechanisms that ensure that the individual immunity of each termite [23]–[27] is not overwhelmed by the pathogen pressure [9]. Among these mechanisms, mutual grooming reduces the cuticular load of pathogens [25], [28]–[31], and burial of cadavers and cannibalism (necrophagy) can prevent pathogens from replicating within the group [22], [32], [33]. When ingested, either by grooming or cannibalism, fungal entomopathogens are inhibited by the gut antimicrobial activity, ultimately preventing fungi from completing their life and disease cycles of replicating and spreading among all individual termites [34], [35].

Although such defense mechanisms may prevent the transfer of pathogens from diseased individuals to healthy individuals [9], it is still possible to kill laboratory groups of subterranean termites by exposing large portions of the group with an artificially high pathogen density [36]–[42]. However, such approaches do not take into account that a diffuse and extended subterranean gallery system [43] makes it virtually impossible to reach most individuals in a field colony with high pathogen densities [10]. Results from the previous studies [36]–[42] imply that there is a threshold ratio of diseased to healthy individuals within a group of termites where behavioral and physiological disease resistance mechanisms cannot prevent the occurrence of the epizootic due to heavy pathogen exposure [10].

**Figure 1:**
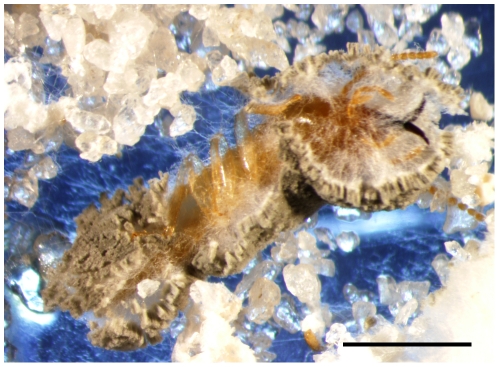
*Metarhizium anisopliae* completing its life cycle, by producing conidia on a dead *Coptotermes formosanus* soldier. Scale bar = 1 mm.

The entomopathogenic fungus *M. anisopliae* has received the most attention for the development of termite biological control and is still perceived by many as a promising candidate for termite control (this erroneous perception was fully discussed in [10]). Chouvenc et al [22] showed that when 6.25% of a group of 960 *Reticulitermes flavipes* (Rhinotermitidae) were exposed to a lethal dosage of *M. anisopliae*, the exposed termites died from the infection, but no secondary infection was observed in the rest of the group after 90 d, because all cadavers were quickly cannibalized. The occurrence of epizootics in a subterranean termite colony mainly depends on the capacity of the pathogenic agent's ability to reach secondary cycling-*i.e.*, to replicate within the termite group and spread among the individuals at a high enough density to continue disease spread in the colony [9]. When introduced in a termite group, *M. anisopliae* should be considered as a semelparous parasite with an obligate killing strategy as described by Ebert and Weisser [44]-*i.e.*, at the individual level, the fungus first needs to kill a termite before it can produce conidia (Fig. 1) for dispersal among other individual termites. This reproductive requirement imposes a limitation on the fungus, because it can only self-replicate and increase its density within the termite nest several days after the death of an infected termite. It may take up to three days post mortem for the fungus to mature and produce conidia [34]. Due to behavioral mechanisms such as cannibalism, burial and avoidance, it was hypothesized that such secondary cycling is unlikely to occur within a termite colony because the cadaver may be removed from the nesting environment before sporulation [9], [22].

This observation leads us to the following question: what is the required proportion of individuals within a termite colony that need to be killed by *M. anisopliae* in order to obtain favorable conditions for secondary spread of the fungus? For the specific case of *M. anisopliae*, we hypothesize that the healthy termites must encounter a cadaver with the fungus producing conidia, at a time when termites can no longer cannibalize or bury the large number of cadavers within their colony. To test this hypothesis, we exposed groups of *Coptotermes formosanus* (Isoptera: Rhinotermitidae) to a sand environment treated with a range of *M. anisopliae* concentrations using planar arenas [45]. Such arenas allowed termite groups to establish their own gallery system and reach a tunnel density equilibrium [46], so as to simulate field conditions as close as possible, in a similar fashion than the “mini-nests” used in ants [47], [48]. The planar arenas provide a sand environment and enabled live termites to bury cadavers, which physically separated the healthy group of termites from the cadavers sporulating with the fungus [45]. Our study showed that, even in groups where termite mortality reached up to 75% due to the fungal pathogen, the surviving termites still had the ability to stop *M. anisopliae* from completing its life cycle within the termite group, thus preventing favorable environmental conditions for an epizootic.

## Results

The 102 experimental arenas with a wide range of conidia density yield termite survivorships ranging from 98% to 0% after 11 d (Dataset S1). The death rate was not affected by the colony of origin (Cox proportional-hazard regression, pairwise comparisons, *p*>0.57) or by the caste (Wald statistic = 0.89, *df* = 1, *p* = 0.37). The density of conidia per gram of sand had a significant effect on termite survivorship when compared with the controls, starting at 1×10^4^ conidia/g, (Wald statistic = 1336, *df* = 1, *p*<0.001) with an average increase of 15% hazard ratio of death per increment of 1×10^4^ conidia/g (*z* = 37.0, *p*<0.001). This result was confirmed by pairwise comparisons of the hazard ratio of death among concentrations (Table 1), and the LD_50_ was estimated at 6.3×10^4^ conidia/g (Dataset S2).

**Table 1:** Termite mortality after 11 d when exposed to *Metarhizium anisopliae* in planar arena.

Conidia per g of sand†	Average mortality (%)	Hazard ratio of death‡	Lower and upper 95% C.I.		Pairwise comparison¤
0 (Control)	5.67	0.107	(0.065	–0.180)	a
2×10^2^	5.67	0.109	(0.065	–0.180)	a
2×10^3^	7.33	0.140	(0.089	–0.221)	ab
5×10^3^	13.67	0.278	(0.196	–0.395)	bc
1×10^4^	13.67	0.274	(0.192	–0.389)	bc
2×10^4^	21.33	0.455	(0.337	–0.614)	cd
3×10^4^	30.00	0.669	(0.511	–0.877)	de
4×10^4^	32.33	0.713	(0.547	–0.929)	de
5×10^4^	42.33	0.976	(0.763	–1.250)	ef
6×10^4^	42.33	1 (Ref)	N	/A	ef
7×10^4^	45.33	1.150	(0.903	–1.460)	fg
8×10^4^	51.00	1.260	(0.994	–1.590)	fg
9×10^4^	57.33	1.600	(1.270	–2.010)	gh
1.0×10^5^	69.00	2.170	(1.740	–2.710)	hi
1.1×10^5^	79.00	2.810	(2.260	–3.490)	ij
1.2×10^5^	87.33	3.790	(3.050	–4.700)	j
2.0×10^5^	95.67	5.360	(4.310	–6.670)	k

† The sand introduced into the arenas was treated with a solution of 0.05% Tween80 containing a suspension of *M. anisopliae* conidia ranging from 0 to 2.0×10^5^ conidia/g.

‡ The hazard ratio of death is displayed using 6×10^4^ c/g (≈LD_50_) as a reference point.

¤ Pairwise comparison of survivorship using a Cox proportional-hazards regression model (α = 0.05, modified with the Holm-Bonferroni method). The same letter indicates no significant difference of ratio of death among treatments (300 termites per treatment).

**Figure 2:**
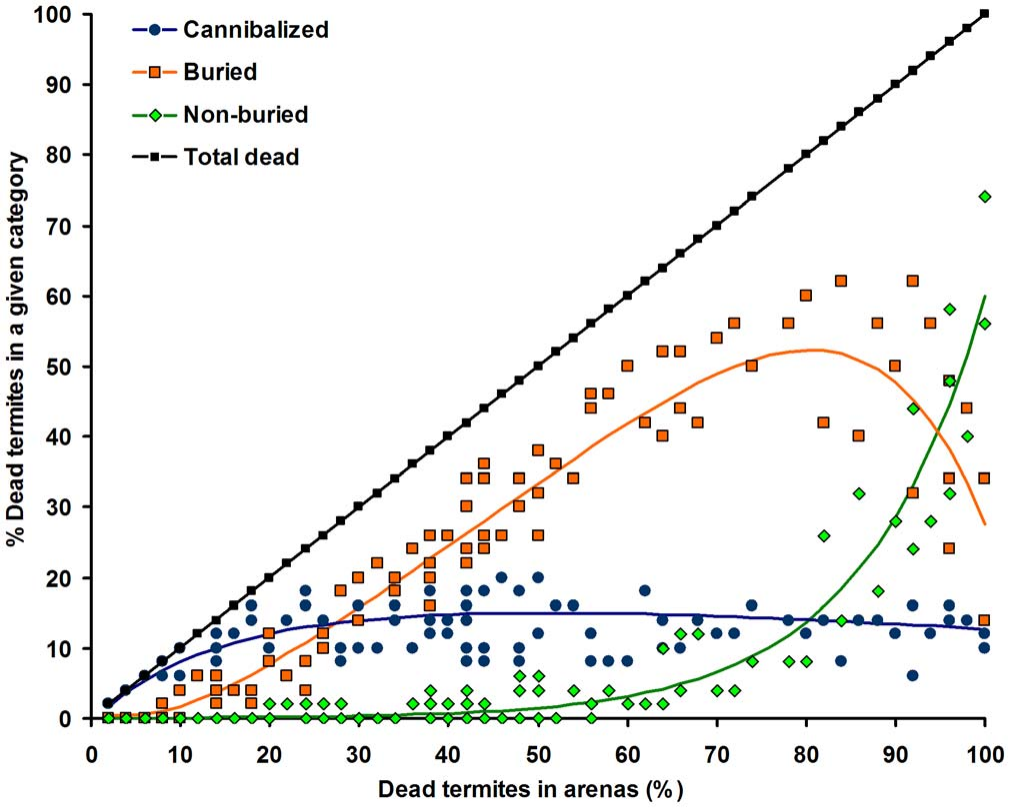
Distribution of dead individuals among three categories: cannibalized, buried and non-buried as a function of the ratio of dead termites within a group. Detailed analysis is in Datasets S3, S4, S5, and S6 in Supporting Information. Superimposed data points are not represented in the figure but were used for the analysis.

**Figure 3:**
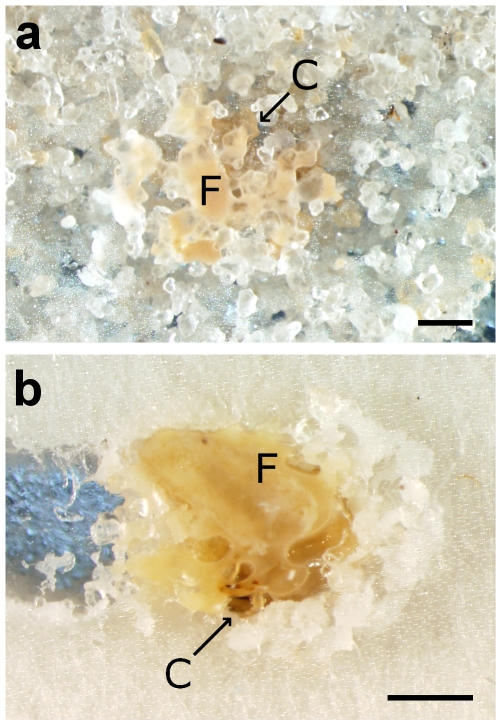
Buried cadavers of *Coptotermes formosanus* workers. In these pictures, the cadavers were covered with fecal material mixed with saliva before the burial. (a) Burial with sand particles. (b) Burial using the cellulose pad as a matrix. Scale bars = 1 mm. C = Cadaver's head, F = Fecal material deposition over the cadaver's abdomen.

For each arena, the number of dead termites was plotted for the three variables (cannibalized, buried, non-buried) in Figure 2. The curve-fitting analysis allowed us to estimate the distribution of dead termites in the three dependant variables, as a function of the death ratio. When the proportion of dead termites in an arena was small (0–15% mortality), most of the dead individuals were cannibalized by their nest mates (Dataset S3). When termite mortality increased with higher densities of conidia, the proportion of cannibalized termites reached a plateau around 15%, as the number of buried termites increased along with the mortality (Dataset S4). Often, cadavers were covered by fecal material from their nest mates before completing the burial (Fig. 3). The proportion of buried termites peaked when the mortality reached 75–80%, but abruptly decreased afterward. Finally, the proportion of non-buried cadavers remained minimal from 0% to 75% mortality, but increased exponentially beyond 75% mortality (Dataset S5). In all arenas combined, out of the 335 dead termites that were not buried, 288 (85.9%) sporulated with *M. anisopliae*, 16 (4.8%) sporulated with *Aspergillus sp.* and 31 (9.3%) showed signs of unidentified bacterial growth.

## Discussion

In our study, a favorable condition for epizootics was defined by the ability for *M. anisopliae* to infect an individual termite and produce conidia after the death of the host, and then to spread within the termite group to infect additional individuals. However, groups of subterranean termites were able to eliminate the cadavers of individuals that had succumbed to the disease by either cannibalizing or burying them, thus preventing the fungal pathogen from replicating within the termite group. Our results showed that when mortality was lower than 15%, cannibalism was the primary means of eliminating cadavers. It was previously shown that ingested *M. anisopliae* is inhibited by the termite's gut antifungal activity [34], [35], which implies that cannibalized cadavers cannot be a source for fungal replication. It was only when the mortality exceeded 15% that dead termites were buried. This suggests that the group of termites was limited in the amount of dead individuals they can cannibalize at any given time, and that burial becomes the alternative way to dispose of the cadavers. Sometimes, before being sealed within the sand, cadavers were deposited with fecal material and saliva. Because termite fecal pellets and saliva possess a strong fungistatic activity [21], [23], [33]–[35], [49], the chance for the fungus to complete its life cycle was reduced. When a buried cadaver was not covered with fecal material, *M. anisopliae* sporulated from the cadaver two to three days after burial. However, even when the fungus successfully completed its life cycle within a buried cadaver by producing a new generation of conidia, these conidia were not able to spread to the rest of the healthy termites due to the physical separation. No buried cadaver was encountered during further tunnel expansion, as previously observed by Li et al. [50], probably due to the avoidance of areas with cadavers [33], [51] that prevented the reopening of the sealed tunnel.

The appearance of sporulating cadavers among live termites occurred only when the mortality from the original exposure reached 75%, and the remaining termites were unable to cannibalize or bury all of the cadavers. Thus, secondary cycling of *M. anisopliae* was only possible when the number of dead termites was too high to allow the survivors to dispose of them. Finally, while it appears already difficult for *M. anisopliae* to complete its life cycle within a termite group, there is also the possibility for saprophytic microorganisms to outcompete *M. anisopliae*, limiting even further its chance to produce conidia, as previously described in ants by Hughes et al [52].

Transmission of the disease agent such as *M. anisopliae* among the termite group is a key for epizootics, as suggested by Pie et al [53]. However, Chouvenc et al [22] demonstrated that the dispersion of *M. anisopliae* within the termite group was not enough to trigger an epizootic even after 90 d because the dispersion of the pathogen lowers its overall concentration which reduces its chance to infect healthy individuals due to a range of defense mechanisms [9]. Therefore, the common assumption that the dispersion of *M. anisopliae* by the social behavior of termites would render biological control of termites possible is inaccurate because the introduced pathogen does not have the chance to establish itself and replicate after the initial dispersion within the colony. The current study demonstrated that in order to bypass the termite colony defense mechanisms, it was necessary to induce at least 75% mortality with one introduction of conidia. This only occurred when the introduced conidia density was at least 1.1×10^5^ conidia/g of sand, which corresponds from two to three orders of magnitude higher than *M. anisopliae* conidia densities usually found in soils [54], [55]. This result raises a dramatic caveat for the potential implementation of fungus, such as *M. anisopliae*, as an introduced biological control agent, because it is virtually impossible to physically reach 75% of individuals in a termite colony with such high densities of conidia. It was previously estimated that only 1% of a subterranean termite colony, which contain up to a million individuals, was directly accessible in monitoring stations [22], [56]. The use of “non-repellent” bait formulations of pathogens was considered to allow for access by most individuals of the colony [57], [58], but no successful formulation was ever developed [10]. Finally, inundative methods were proved inefficient against subterranean termites due to their extensive tunnel system [10], [43]. Therefore, exposing the majority of a subterranean termite colony to a lethal dose of *M. anisopliae* remains an unsolved problem.

A pathogen that does not require an obligate killing strategy for completing its life cycle would bypass some aspects of the multilevel resistance in subterranean termites [9]. Unfortunately, the bulk of the termite biological control research focused on the use of pathogens with an obligate killing strategy such as *M. anisopliae* or entomopathogenic nematodes. Termite-virus associations remain to be examined in a meaningful way, and no bacterial strain has been found to be both non-repellent and virulent at biologically relevant concentration [10]. It is our hope that some of the resources that continue to be spent on fungus-based biological control will be turned toward a better understanding of alternative pathogens.

Although *M. anisopliae* may be virulent pathogenic fungus to termites at the individual level, it does not have the capability to trigger an epizootic at the colony level. Social insect colonies have been considered superorganisms [59], [60] in which the social immunity was compared by analogy to the individual immunity of higher vertebrates [61]. Following this analogy, where each individual termite is a “cell” of the superorganism and taking our results into consideration, *M. anisopliae* can only suppress a small proportion of “cells” at a time, and therefore has a limited impact on the overall colony health. Thus, at the termite colony level, *M. anisopliae* acts as a relatively avirulent parasite, an organism that uses a host as a resource for completing its life cycle, without killing its host (here, the termite colony). This analogy implies that in a subterranean termite colony, *M. anisopliae* expresses a multilevel virulence that depends on the level of organization of the host. By establishing disease resistance mechanisms at multiple levels of organization within the colony [9] and producing environmental conditions unfavorable to fungal replication, subterranean termites, as a colony, challenge the traditional concepts of epizootics, by manipulating the level of virulence of fungal parasites. This explains why biological control of termites with such introduced fungal entomopathogens has failed so far [10], [62].

In order to increase the chances for a fungal entomopathogen to reach secondary cycling within the termite colony, alternative approaches are required. Recent studies [9], [26], [63], [64] suggested that in order to have successful biological control, methods to allow pathogens to bypass the termites' multilevel disease resistance mechanisms or to render the termite nest environment favorable to the pathogen's secondary cycling are needed. Although a comprehensive theoretical framework was established by these authors, the transformation of such ideas into realistic application remains challenging [10].

## Materials and Methods

### Termite Preparation

Termites were collected from three field colonies of the Formosan subterranean termite *C. formosanus* in Fort Lauderdale, FL using the method described by Su and Scheffrahn [65], processed in the laboratory according to Tamashiro et al [66] and kept in groups of at least 5,000 for 10–15 d in containers at 28°C before use in the bioassay. No specific permits were required for the termite's collection from field colonies. For each colony, groups of 50 termites were prepared, using a caste ratio of 45 workers (undifferentiated larvae of at least the third instar) and five soldiers. The groups of termites were then introduced into a planar arena [45]. In our study, the planar arena has the advantage over other standard protocols (Petri dish, jars) because it provides a soil environment enabling termites to forage and establish their own tunnel structure, while allowing continuous monitoring.

### Arena Setup

**Figure 4:**
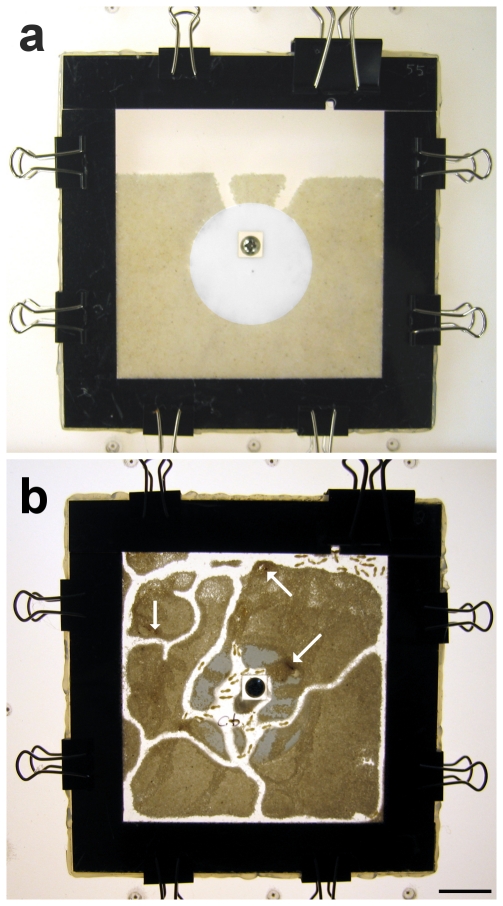
Planar arena for the termite bioassay. (a) Planar arena setup before introducing the termites, adapted from Chouvenc et al [45]. The space between the two sheets of Plexiglas remained even throughout the arena due to the central screw-spacer elements. Termites were introduced through the top-right hole before closing. (b) Example of a planar arena used for the bioassay at day 11. Termites established a gallery system and fed upon the cellulose pad. Arrows show areas where dead termites were buried. Scale bar = 2 cm.

Arenas were composed of two sheets of transparent Plexiglas (12 by 12 by 0.2 cm in thickness) separated from each other by Plexiglas laminates (2 cm in width and 0.2 cm in thickness) on the four sides, creating a 10 by 10 by 0.2 cm space inside the arena. A 0.8 by 0.8 by 0.2 cm spacer was placed in the center of the arena. The two sheets of Plexiglas and the central spacer were held together by a 3 mm-diameter screw, in order to maintain the 0.2 cm space layer evenly throughout the entire arena. A 0.4 mm diameter hole was drilled on the top Plexiglas sheet, 1 cm away from the spacer-screw hole, for adding liquids with the help of a syringe and to allow a small amount of air flow. A 5-mm-diameter hole was provided in one corner for the introduction of the termites into the arena chamber. Before assembly, all elements were washed with soap, immersed in bleach (3% sodium hypochlorate solution) for 2 h and rinsed three times with sterile deionized water. A sterile cellulose absorbent pad (45 mm in diameter, 2 mm thick), prepared by puncturing a 0.8 by 0.8 cm hole, was placed in the arena, fitting in with the spacer and centered with the 0.4 mm hole. The arena was filled with 18 g of wet sand (15 g of sterile dry sand, 150–500 µm size, and 3 ml of treatment solution, see below), leaving a band of 10 by 2.5 cm empty on one border, exposing the absorbent pad to the empty space. The arena pieces were kept together with eight 1-cm binder clips and the mounted arena was set horizontally (Fig. 4A). The four sides of the arena were sealed by hot glue, in order to prevent sand desiccation. One ml of sterile deionized water was injected via the 0.4 mm center hole onto the absorbent pad. A group of 50 termites was introduced into the arena with the help of a small funnel, and the introduction hole was sealed with a thin transparent plastic cover after all termites entered the arena.

### Soil Treatments

The sand introduced in the planar arena was previously treated with a suspension of fungal conidia. A 10^8^ conidia/ml stock solution of *M. anisopliae* (strain ATCC 90448) was prepared as described in Chouvenc et al [22]. Due to the hydrophobic nature of the conidia, a 0.05% Tween80 aqueous solution was used for the stock conidia suspension. The conidia concentrations were adjusted in a 0.05% Tween80 solution, and 3 ml of the appropriate concentrations were mixed homogenously with 15 g of dry sterile sand to obtain final densities of 0 (control, 0.05% Tween80), 2×10^2^, 2×10^3^, 5×10^3^, 1×10^4^, 2×10^4^, 3×10^4^, 4×10^4^, 5×10^4^, 6×10^4^, 7×10^4^, 8×10^4^, 9×10^4^, 1×10^5^, 1.1×10^5^, 1.2×10^5^ and 2×10^5^ conidia per g of dry sand. Six replicates per conidia density were prepared (two replicates per termite colony of origin) for a total of 102 arenas using 5,100 termites.

For confirmation of accurate densities of conidia in the sand for all arenas, a subsample of 0.6 g wet sand was removed from each individual arena before sealing. These subsamples were subjected to serial dilutions and plated on 1/5 strength potato dextrose agar to determine germination after 2 d. If the number of fungal colony forming units (CFU) was ±5% beyond the expected density, the arena was removed from the experiment, and replaced with a new replicate with the correct conidia density.

Arenas were placed at 28°C in the dark and the termite mortality was monitored daily for 11 d by temporarily illuminating the arenas from below with LED lights and by taking digital pictures (Fig. 4B). Dead termites were not removed. At 11 d, final mortality was recorded and dead termites were classified in three categories: cannibalized, buried, and non-buried. Cannibalized termites were determined by subtracting from the original number of termites (50) the number of live termites, the number of buried termites and the number of non-buried dead termites. For dead termites that were not buried, fungal sporulation was assessed at day 11 and again at day 14, to allow enough time for recent cadavers to sporulate and confirm *M. anisopliae* completion of life cycle.

### Statistical Analysis

To determine the termite susceptibility to the fungus, the median lethal dosage (LD_50_) was determined using a Probit analysis [67]. A Cox proportional-hazard regression analysis (using the program R-Project for statistical computing, version 2.4; http://cran.r-project.org/) was performed on all the individuals and a Wald statistic was generated. The resulting hazard function defines the instantaneous rate of death at a particular time, while controlling the effects of other variables on survival. Pairwise comparisons of the death rates among fungal conidia densities were adjusted by the Holm-Bonferroni method (α = 0.05). The effect of colony of origin and castes on the survivorship were also tested through a Cox proportional-hazard regression analysis.

After the numbers of dead termites that were cannibalized, buried and non-buried were determined at 11 d in all 102 arenas, each value was plotted with “total number of dead termites” as the independent variable and “cannibalized” “buried”, and “non-buried” numbers as dependent variables. Because the three latter variables are dependent on each other, curve fitting analysis for their theoretical distribution was determined with this additional requirement: 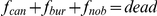, where 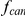 represents the number of cannibalized termites as a function of the total number of dead termites, 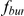 represents the number of buried termites as a function of the total number of dead termites, 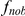 represents the number of non-buried termites as a function of the total number of dead termites, and 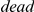 represents the total number of dead termites (Dataset S6). The goodness-to-fit of the three theoretical distributions to the empirical data was analyzed by using Pythonequations 25.4 (http://code.google.com/p/pythonequations ). A F-Statistic was provided for each multi-variate regression by using Rpy (http://rpy.sourceforge.net/).

## Supporting Information

Dataset S1 **Survivorship of groups of 50 termites in arenas filled with sand treated with** ***Metarhizium anisopliae*** **conidia.**
(PDF)

Dataset S2 **Probit analysis of termite's mortality at day 11 after introduction into the arenas.**
(PDF)

Dataset S3 **Distribution of buried termites in function of the number of dead termites at 11 d.**
(PDF)

Dataset S4 **Distribution of buried termites in function of the number of dead termites at 11 d.**
(PDF)

Dataset S5 **Distribution of non-buried termites in function of the number of dead termites at 11 d.**
(PDF)

Dataset S6 **Confirmation of the validity of the curve fitting analysis.**
(PDF)
