# Supplementary material for: When Subterranean Termites Challenge the Rules of Fungal Epizootics
Source: PLoS One. 2012 Mar 28;7(3):e34484. doi: 10.1371/journal.pone.0034484 (PMC3314638; doi:10.1371/journal.pone.0034484)

Dataset S1. Survivorship of groups of 50 termites in arenas filled with sand treated with *Metarhizium anisopliae* conidia. **A:** Survivorship from all of the 102 arenas. **B:** Average survivorship per treatment (densities are conidia/g of sand). Significant differences of survivorship among treatments are described in Table 1.

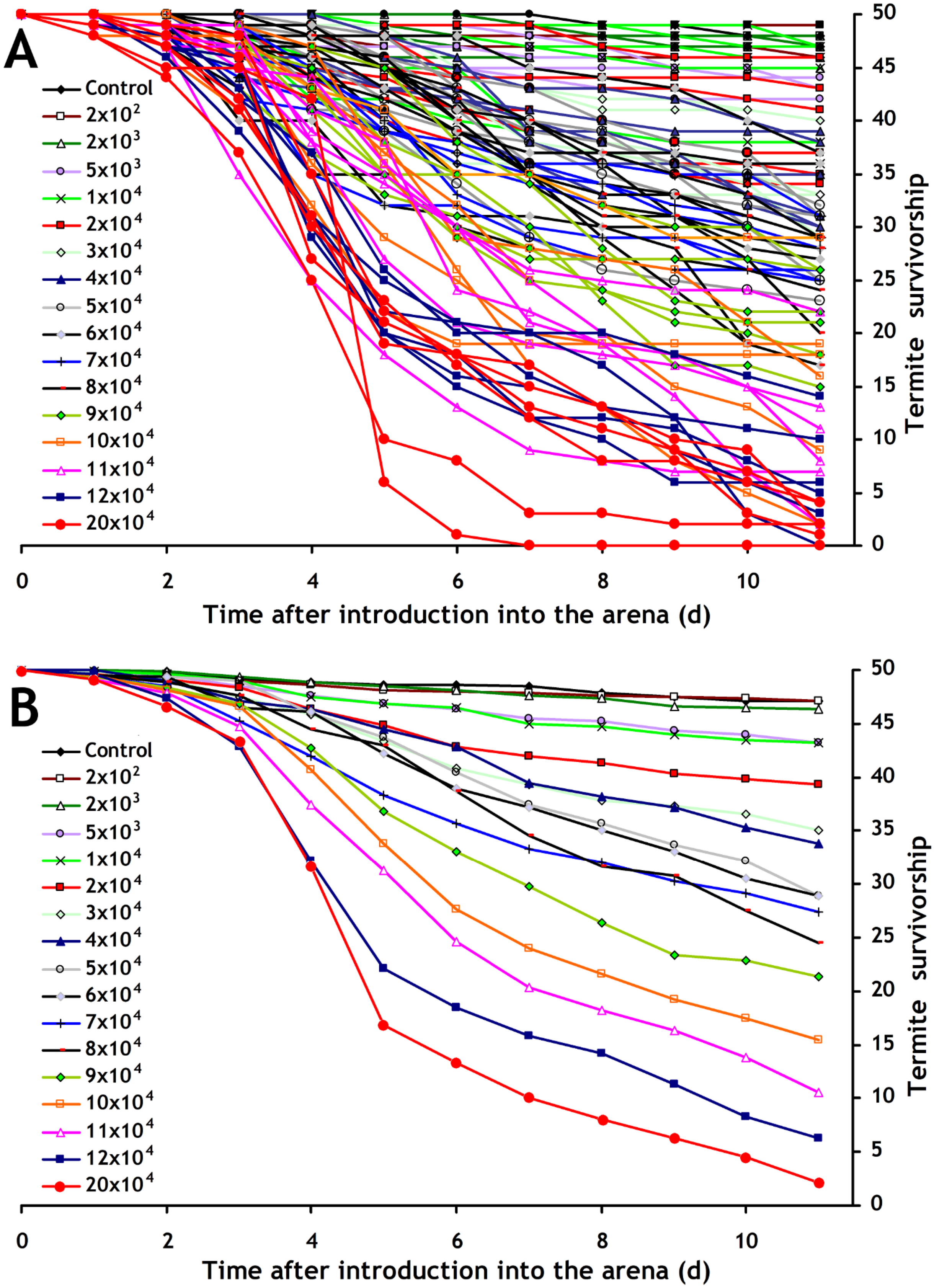

Supplement: Dataset S1 — Survivorship of groups of 50 termites in arenas filled with sand treated with Metarhizium anisopliae conidia. (PDF) [file pone.0034484.s001.pdf]
