# Supplementary material for: When Subterranean Termites Challenge the Rules of Fungal Epizootics
Source: PLoS One. 2012 Mar 28;7(3):e34484. doi: 10.1371/journal.pone.0034484 (PMC3314638; doi:10.1371/journal.pone.0034484)
Supplement: Dataset S2 — Probit analysis of termite's mortality at day 11 after introduction into the arenas. (PDF) [file pone.0034484.s002.pdf]

Dataset S2. Probit analysis of termite's mortality at day 11 after introduction into the arenas

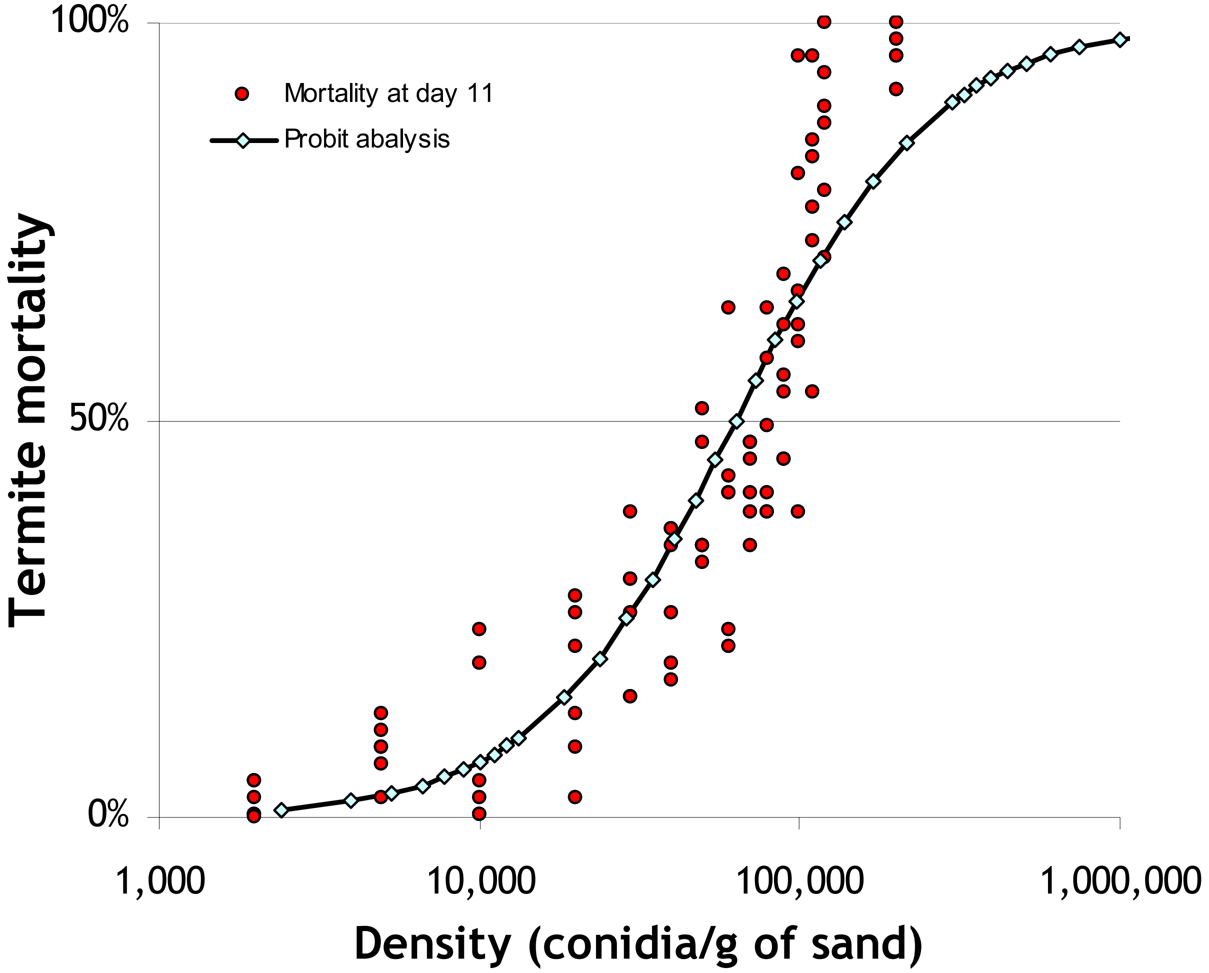

Probit analysis:Type III Analysis of Effects

Effect	DF	WaldChi-Square	Pr > ChiSq
Log10(Dose)	1	1508.4904	<0.0001

Analysis of Parameter Estimates

Parameter	DF	Estimate	Standard error	95% confidence limit		Chi-Square	Pr> ChiSq
Intercept	1	-15.568	0.401	-16.355	-14.781	1503.74	<0.0001
Log10(dose)	1	3.242	0.083	3.079	3.4064	1508.49	<0.0001

Probit Model in Terms of Tolerance Distribution

Mu	Sigma
4.80084	0.30837

Estimated Covariance Matrix for Tolerance Parameters

	Mu	Sigma
Mu	0.000060	0.000000
Sigma	0.000000	0.000063
