# Supplementary material for: When Subterranean Termites Challenge the Rules of Fungal Epizootics
Source: PLoS One. 2012 Mar 28;7(3):e34484. doi: 10.1371/journal.pone.0034484 (PMC3314638; doi:10.1371/journal.pone.0034484)
Supplement: Dataset S3 — Distribution of buried termites in function of the number of dead termites at 11 d. (PDF) [file pone.0034484.s003.pdf]

Dataset S3. Distribution of cannibalized termites as function of the number of dead termites at 11 d.

$$f_{can} = a + b\times ln(ex+f) + c\times ln(ex+g)^2$$

a = -99.4979340
b = 40.1511811
c = -3.76595000
e = 6.20952430
g = 48.1261494

Note: Coefficients for the curve fitting analysis are from groups of 50 termites (not percentages, as presented in the graphs)

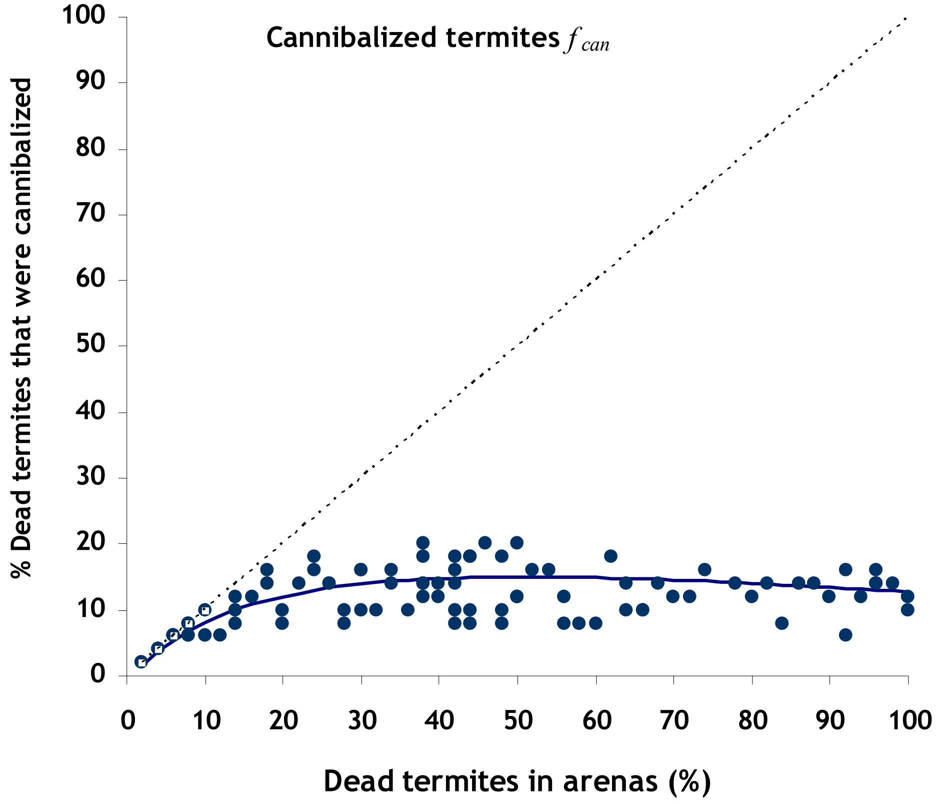

Fitting target of sum of squared absolute error = 248.67546

Fit statistics

Degrees of freedom (error): 97
Degrees of freedom (regression): 4
R-squared: 0.49232
R-squared adjusted: 0.47139
Model F-statistic: 23.5170
Model F-statistic p-value: $1.30784e^{-13}$
Model log-likelihood: -190.18170
AIC: 3.82709
BIC: 3.95577
RMSE: 1.56140

	Absolute error
Minimum:	-2.65790
Maximum:	3.65507
Mean:	0.33283
Std. Err. of Mean:	0.15179
Median:	-0.15179
Variance:	2.32721
Std. Deviation:	1.52552
Pop. Var.:	2.32721
Pop. Std. Dev.:	1.52552
Skew:	0.42162
Kurtosis:	-0.42111
