# Supplementary material for: When Subterranean Termites Challenge the Rules of Fungal Epizootics
Source: PLoS One. 2012 Mar 28;7(3):e34484. doi: 10.1371/journal.pone.0034484 (PMC3314638; doi:10.1371/journal.pone.0034484)
Supplement: Dataset S4 — Distribution of buried termites in function of the number of dead termites at 11 d. (PDF) [file pone.0034484.s004.pdf]

Dataset S4. Distribution of buried termites as function of the number of dead termites at 11 d.

$$f_{bur} = a + bx^1 + cx^2 + ex^3 + gx^4 + hx^5$$

a = 0.445373433
b = -0.3305326422
c = 0.1031001398
e = -0.0045182294
g = 0.000103013
h = -0.0000009.82

Note: Coefficients for the curve fitting analysis are from groups of 50 termites (not percentages, as presented in the graphs)

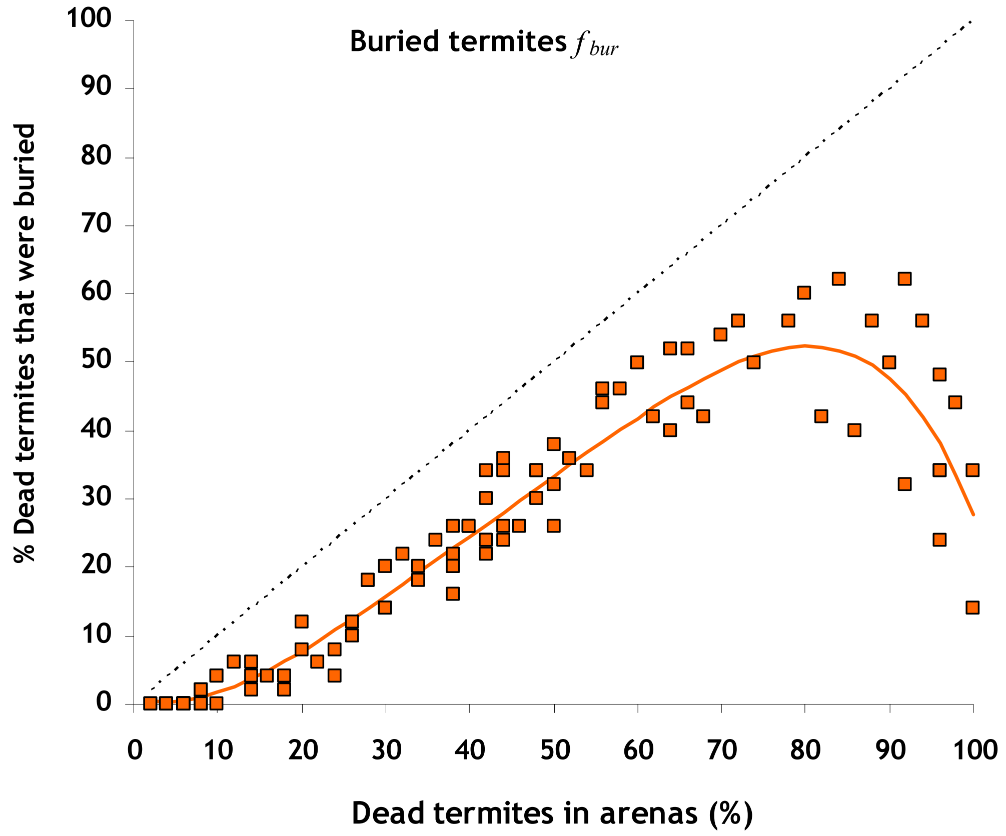

Fitting target of sum of squared absolute error = 705.81526

Fit statistics

Degrees of freedom (error): 96
Degrees of freedom (regression): 5
R-squared: 0.92355
R-squared adjusted: 0.92355
Model F-statistic: 231.95468
Model F-statistic p-value: $<1e^{-16}$
Model log-likelihood: -243.38514
AIC: 4.88990
BIC: 5.04431
RMSE: 2.63054

	Absolute error
Minimum:	-8.40667
Maximum:	7.05882
Mean:	-0.22155
Std. Err. of Mean:	0.26081
Median:	-2.45580
Variance:	6.87067
Std. Deviation:	2.62119
Pop. Var.:	6.87067
Pop. Std. Dev.:	2.62119
Skew:	-0.00318
Kurtosis:	1.18913
