# Supplementary material for: When Subterranean Termites Challenge the Rules of Fungal Epizootics
Source: PLoS One. 2012 Mar 28;7(3):e34484. doi: 10.1371/journal.pone.0034484 (PMC3314638; doi:10.1371/journal.pone.0034484)
Supplement: Dataset S5 — Distribution of non-buried termites in function of the number of dead termites at 11 d. (PDF) [file pone.0034484.s005.pdf]

Dataset S5. Distribution of non-buried termites as function of the number of dead termites at 11 d.

$$f_{nob} = a \times exp(bx)$$

a = 0.019041202
b = 0.147230859

Note: Coefficients for the curve fitting analysis are from groups of 50 termites (not percentages, as presented in the graphs)

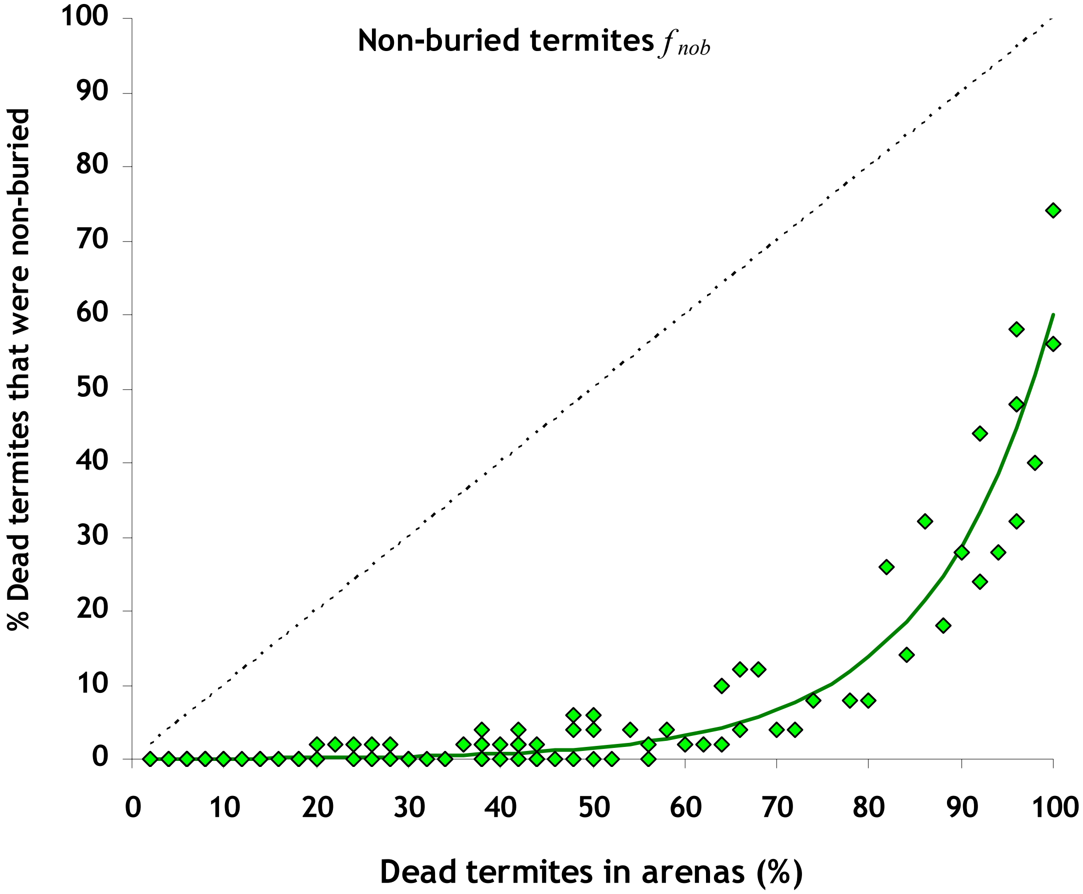

Fitting target of sum of squared absolute error = 409.28397

Fit statistics

Degrees of freedom (error): 100
Degrees of freedom (regression): 1
R-squared: 0.92389
R-squared adjusted: 0.92313
Model F-statistic: 1213.95734
Model F-statistic p-value: $<1e^{-16}$
Model log-likelihood: -215.59292
AIC: 4.26652
BIC: 4.31799
RMSE: 2.00314

	Absolute error
Minimum:	-7.02412
Maximum:	6.33000
Mean:	-0.12324
Std. Err. of Mean:	0.19894
Median:	-0.03431
Variance:	3.99740
Std. Deviation:	1.99935
Pop. Var.:	3.99740
Pop. Std. Dev.:	1.99935
Skew:	0.33328
Kurtosis:	3.89085
