# Supplementary material for: When Subterranean Termites Challenge the Rules of Fungal Epizootics
Source: PLoS One. 2012 Mar 28;7(3):e34484. doi: 10.1371/journal.pone.0034484 (PMC3314638; doi:10.1371/journal.pone.0034484)
Supplement: Dataset S6 — Confirmation of the validity of the curve fitting analysis. (PDF) [file pone.0034484.s006.pdf]

Dataset S6. Confirmation of the validity of the curve fitting analysis.

The curve fitting analysis was considered valid for the three dependent variables if the following requirement was fulfilled, where ***dead*** is the total number of dead termites:

$$f_{can} + f_{bur} + f_{nob} = \boldsymbol{dead}$$

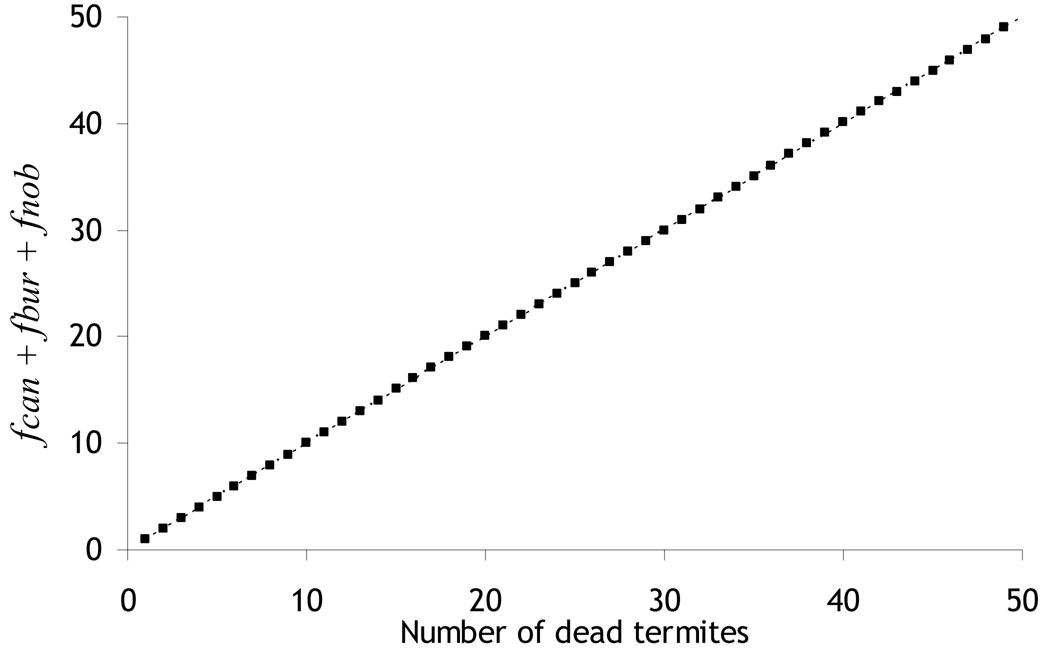

Fitting target of sum of squared absolute error = 0.18440

Fit statistics

Degrees of freedom (error): 49
Degrees of freedom (regression): 1
R-squared: 0.99998
R-squared adjusted: 0.99998
Model F-statistic: 2710347.21296
Model F-statistic p-value: $<1e^{-16}$
Model log-likelihood: 69.119248
AIC: -2.68476
BIC: -2.60828
RMSE: 0.06072

	Absolute error
Minimum:	-0.19635
Maximum:	0.12546
Mean:	$-1.752e^{-08}$
Std. Err. of Mean:	0.00867
Median:	0.00185
Variance:	0.00369
Std. Deviation:	0.06072
Pop. Variance:	0.00369
Pop. Std. Dev.:	0.06072
Skew:	-0.37649
Kurtosis:	0.60947

$f_{can} + f_{bur} + f_{nob} = \boldsymbol{dead}$ at all given points, thus the curve fitting analysis was validated.
